# The effects of host plant species and larval density on immune function in the polyphagous moth *Spodoptera littoralis*

**DOI:** 10.1002/ece3.7802

**Published:** 2021-07-15

**Authors:** Kristina Karlsson Green

**Affiliations:** ^1^ Department of Plant Protection Biology Swedish University of Agricultural Sciences Alnarp Sweden

**Keywords:** crop protection, ecological immunology, host plant performance, Lepidoptera, nutritional immunology, phenotypic plasticity

## Abstract

Immune functions are costly, and immune investment is usually dependent on the individual's condition and resource availability. For phytophagous insects, host plant quality has large effects on performance, for example growth and survival, and may also affect their immune function. Polyphagous insects often experience a large variation in quality among different host plant species, and their immune investment may thus vary depending on which host plant species they develop on. Larvae of the polyphagous moth *Spodoptera littoralis* have previously been found to exhibit density‐dependent prophylaxis as they invest more in certain immune responses in high population densities. In addition, the immune response of *S. littoralis* has been shown to depend on nutrient quality in experiments with artificial diet. Here, I studied the effects of natural host plant diet and larval density on a number of immune responses to understand how host plant species affects immune investment in generalist insects, and whether the density‐dependent prophylaxis could be mediated by host plant species. While host plant species in general did not mediate the density‐dependent immune expression, particular host plant species was found to increase larval investment in certain functions of the immune system. Interestingly, these results indicate that different host plants may provide a polyphagous species with protection against different kinds of antagonisms. This insight may contribute to our understanding of the relationship between preference and performance in generalists, as well as having applied consequences for sustainable pest management.

## Graphical abstract

The effects of host plant species and larval density are studied on a number of immune responses in the polyphagous moth *Spodoptera littoralis* to understand whether host plant species affects immune investment and whether the density‐dependent prophylaxis could be mediated by host plant species. While host plant species in general did not mediate the density‐dependent immune expression, particular host plant species was found to increase investment in certain functions of the immune system. These results indicate that different host plants may provide a polyphagous species with protection against different kinds of antagonisms, which may contribute to our understanding of the relationship between preference and performance in generalists, as well as having applied consequences for sustainable pest management.

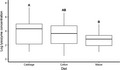

## 1 INTRODUCTION

The immune system is a costly trait, and there are thus often trade‐offs between investment in an increased immune response and other traits (Lochmiller & Deerenberg, 2000; Schwenke et al., 2016), such as between immune function and reproduction (Ilmonen et al., 2000), or growth and intraspecific competition (Kraaijeveld & Godfray, 1997). As a consequence, immune functions are often condition‐dependent and individuals are expected to only invest in immunity when they are infected or under risk of infection. To mediate trade‐offs in resource allocation, the dietary nutrient content is of crucial importance (Ponton et al., 2011, 2013). The importance of diet for immune functions has been demonstrated in various animals (e.g., birds (Birkhead et al., 1999; McGraw et al., 2006), mammals (Shaner et al., 2018), reptiles (French et al., 2007; Holden et al., 2019), and insects (Alaux et al., 2010; Miller & Cotter, 2018; Ponton et al., 2020)), where immune functions could be improved both by diet quantity and quality. The resource environment that individuals experience in nature may thus affect their possibility to increase immune investment.

For phytophagous insects, the host plant provides larval insects with nutrients of varying quality and quantity but also with challenges in terms of defensive chemical compounds (Behmer, 2009; Schoonhoven et al., 2005). The host plant has therefore large impact on larval performance and survival (e.g., Coley et al., 2006) and may also affect their immune function (Singer et al., 2014). Although most studies on the effects of diet on insect immune function have been done by manipulating artificial diet content (e.g., Barthel et al., 2016), research has, for example, shown that secondary metabolites (Laurentz et al., 2012) or different plant varieties (Vogelweith et al., 2013) may affect immune investment in insects. Several studies on how the host plant affects insect immune function have been made on specialist insects (Carper et al., 2019; del Campo et al., 2013; Kelly & Bowers, 2018; Smilanich et al., 2009, but see, for example, Muller et al., 2015, for an example from a generalist species), perhaps because they often are adapted to the specific chemistry of their particular host plant species. Generalist insects, on the other hand, may not be as good in utilizing species‐specific plant chemicals but should instead be able to tolerate and develop on a wide range of host plants that may be very different in quality and in how they affect insect performance (Rothwell & Holeski, 2020). The resource environment for generalist species could therefore be very diverse with good performance on some host plant species and poor development on others (e.g., Gómez Jiménez et al., 2014; Shikano et al., 2010; Tikkanen et al., 2000). Given that immune function studies with manipulated artificial diet have shown that differences in nutrient content could have large effects on various immune parameters (e.g., Ponton et al., 2020), the host plant diversity of both nutrients and toxic defenses that generalist insects are exposed to present excellent opportunities to study the effect of natural diet variation for immune responses (Singer et al., 2014).

The polyphagous moth *Spodoptera littoralis* is a model species for studies on insect immune function (Cotter et al., 2004, 2008, 2011; Cotter et al., 2004; Cotter & Wilson, 2002; Lee et al., 2006, 2008). This species exhibits density‐dependent phase polyphenism, which is a case of phenotypic plasticity (see e.g., West‐Eberhard, 2003) where the expressed phenotype depends on the population density that the individual experiences (Applebaum & Heifetz, 1999). As other *Spodoptera species*, *S. littoralis* larvae increase their degree of cuticular melanization in crowded conditions (Wilson et al., 2001). Melanization is a wide‐spread process among animals, which could have several different functions such as thermo‐regulation and protection against UV radiation (San‐Jose & Roulin, 2018). In addition, melanization is in general involved in immune functions as it provides individuals with both internal and external protection, for example, by encapsulating foreign particles (San‐Jose & Roulin, 2018). The phase‐polyphenic melanization in *S. littoralis* has therefore been suggested to be a density‐dependent prophylaxis where larvae invest in immune function in crowded conditions where there is a higher risk for pathogen transfer between individuals (Wilson & Reeson, 1998).

Research has shown that *S. littoralis* larvae indeed increase investment of some immune parameters in higher densities, but not in others. For example, Cotter, Hails, et al. (2004) found that the high‐density phenotype had increased activity of the enzyme phenoloxidase (PO), which is an important feature of insect immune system as it catalyzes melanin production (Gonzalez‐Santoyo & Cordoba‐Aguilar, 2012) and protects against fungal infections (Dubovskiy et al., 2013). On the contrary, high‐density phenotypes had lower lysozyme activity, which is an antibacterial defense (Kurtz et al., 2000). Beside density, immune investment in *S. littoralis* is affected by dietary nutrient content (Cotter et al., 2011; Lee et al., 2008). To my knowledge, however, all studies hitherto have been performed with artificial diet and it has previously not been studied how different host plant species may affect immune response in *S. littoralis*.

*Spodoptera littoralis* feeds on more than 80 different plant species from over 40 different families (CABI, 2019), and the host range thus spans over plants which may be very different in terms of nutrient content and chemical defenses. This species has been shown to be very plastic in terms of host plant preference and performance (Anderson et al., 2013; Lhomme et al., 2018; Proffit et al., 2015; Rösvik et al., 2020; Roy et al., 2016; Thöming et al., 2013), where larval development, fecundity, and mating propensity differ depending on which host plant species they feed on (Rösvik et al., 2020; Karlsson Green et al., 2021). Furthermore, parasitoid success on *S. littoralis* could differ depending on host plant species, which may result from a combination between increased larval immune defense and variation in parasitoid search behavior depending on plant species (Sadek et al., 2010). Here, I studied the effects of host plant species, larval density, and their interaction on different immune responses in *S. littoralis,* asking whether plant species has the potential to mediate the prophylactic investment in immune function and whether certain plant species are more advantageous for the immune system than others.

## 2 MATERIALS AND METHODS

### 2.1 Study species and rearing

*Spodoptera littoralis* is a severe crop pest with a geographic distribution across Africa and in local areas in Southern Europe (CABI, 2019). Ovipositing females choose a host plant depending on innate preferences and their larval host plant experience (Anderson et al., 2013; Lhomme et al., 2018; Thöming et al., 2013) and the hatching offspring develop and feed on the selected host plant during six instars before they pupate in the soil. For the current experiments, a laboratory population of *S. littoralis*, that originates from Egypt and is being maintained at SLU Alnarp, was used.

As larval host plants, greenhouse‐grown cotton (*Gossypium hirsutum*), cabbage (*Brassica oleracea* v. *capitata*), and maize (*Zea mays*) were used. These species are all present in Egypt where the *S. littoralis* laboratory population here used originates from. Research has shown that while ovipositing females prefer cotton and maize over cabbage, irrespective of previous experience of cabbage (Thöming et al., 2013), larval performance is generally superior on cotton and cabbage and very poor on maize (Rösvik et al., 2020).

To study the effects of host plant species and larval density on immune investment, a diet experiment was performed where larvae were reared on detached leaves of the three different plant species. Newly hatched larvae were reared in groups in plastic boxes (H*W*L 6.5*18*22 cm) and fed either cotton, cabbage, or maize, until the 2nd instar when random larvae were gently transferred with a brush to plastic cups of 1.0 dl volume. Either one larva—the low‐density treatment—or four larvae—the high‐density treatment—were transferred to each cup and thereafter fed with detached leaves until they reached the last instar, when the immune assays were performed. To avoid pseudo‐replication, only one of the four larvae in each cup of the high‐density treatment was sampled. All treatments were given ad libitum food, and larvae were reared in climate chambers with controlled settings of 16:8 L:D, 25°C, 60% RH. In total, 128 larvae were studied in the immune assays of which 44 were fed cabbage, 49 cotton, and 35 maize in either high or low density (in total 62 larvae in high density and 66 larvae in low density). However, since not all assays were possible to perform with all larvae, the sample size between immune assays differs (N ranges between 100–128).

### 2.2 Cuticular melanization and artificial encapsulation

**FIGURE 1:**
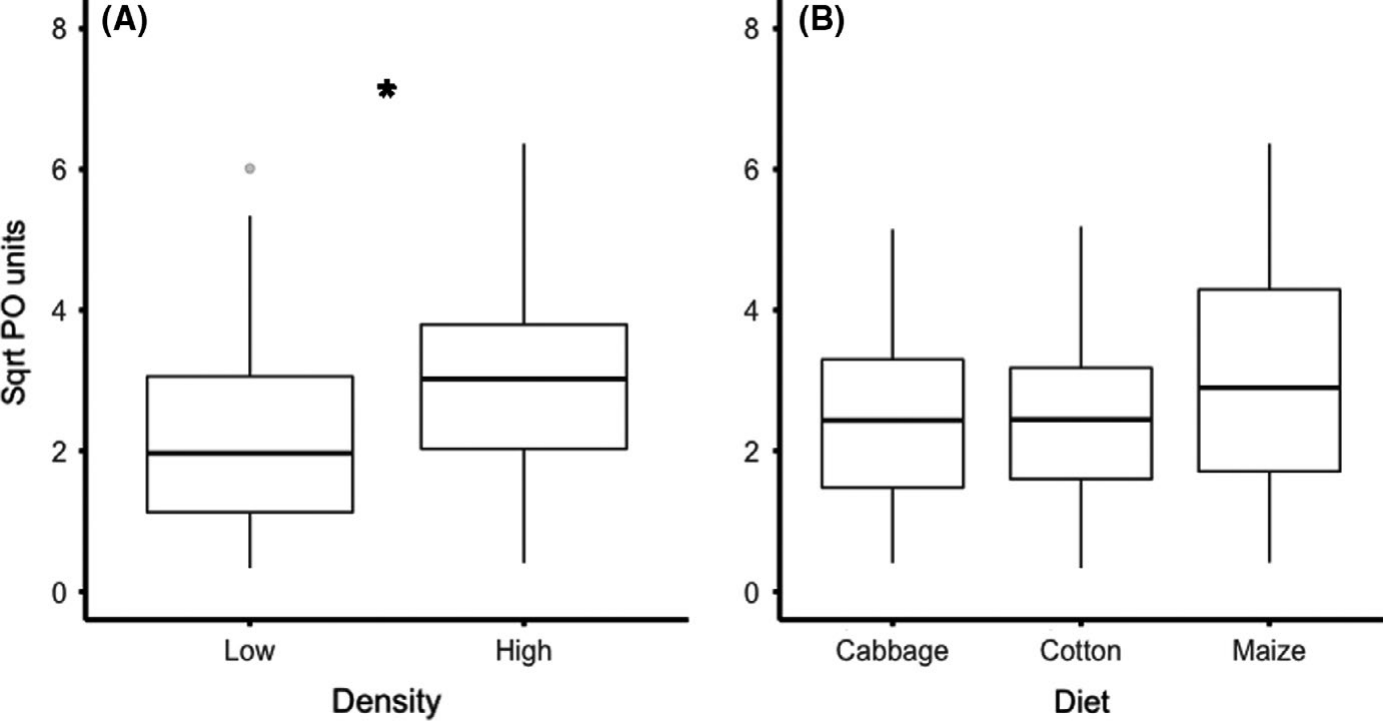
The effects of larval density and host plant diet on hemolymph PO activity. (a) The significant difference between low and high‐density larvae in PO activity and (b) the tendency (*p* = .054) for differences between cabbage, cotton, and maize fed larvae in PO activity. Boxes indicate 25th and 75th percentiles, lines within boxes indicate medians, and whiskers includes values within 1.5 times the interquartile range. An asterisk (*) indicate significant differences between treatments (*p* < .05), results from general linear model (Table 1), *N* = 117

To study the degree of cuticular melanization, larval color was scored by eye using a reference picture provided in Figure 1 by Cotter et al. (2008) with seven steps ranging from 1 (very pale) to 7 (very black). This scale has previously been found to correlate with quantitative spectrometer recording and is thus a reliable approach to measure cuticular melanization in *S. littoralis* (Cotter et al., 2008).

To measure the ability of larvae to withstand parasitoids, their ability to encapsulate a nylon piece in a simulated parasitoid attack was measured. First, a larva was punched with a thin needle between the last of the left‐side prolegs and hemolymph was collected on ice to use for the PO, protein, and lysozyme analyses (see below). Thereafter, a 2 mm long piece of nylon thread was gently inserted in the hole, sliding in under the skin, and left during 24 hr. After 24 hr, the nylon filament was removed and stored in a freezer until it was photographed. Each piece was photographed twice, and the encapsulated area as well as the degree of the melanization, measured as gray values, were recorded with the software ImageJ. The average between the two photographs of each nylon piece was used for statistical analyses.

### 2.3 Phenoloxidase activity and protein content

To measure the degree of PO activity, hemolymph was collected as above and stored undiluted in −80°C freezer until further analyses. To analyze the samples, they were thawed and 4 µl hemolymph was added to 200 µl PBS before being vortexed. Two replicates of each sample were prepared by adding 90 µl of dopamine to 90 µl of the buffered hemolymph. The samples were then analyzed on a SpectroStar Nano plate reader where PO activity was measured spectrophotometrically at 492 nm and 25°C during the first 30 min, which is in the linear phase of the reaction. The activity was measured in PO units, where one unit is equivalent to the amount of enzyme that increases absorbance with 0.001 per minute.

The hemolymph protein content was measured using the Bio‐Rad protein assay kit with BSA as standard (Bio‐Rad Laboratories). Two replicates of 5 µl of the PBS buffered hemolymph were analyzed with SpectroStar Nano plate reader at 600 nm.

### 2.4 Lysozyme activity

To measure the lysozyme activity in the hemolymph, a lytic zone assay was performed. Agar plates of 10 ml, containing 5 mg per ml of the freeze‐dried bacteria *Micrococcus lysodeikticus* (Sigma‐Aldrich), were prepared as in Kurtz et al. (2000). Approximately 20 holes of 2 mm each were punched in the agar, and 1 µl of undiluted hemolymph was pipetted into each hole, two replicates of each sample. A standard series of hen egg white lysozyme was also analyzed in the agar plates. The agar plates were incubated at 33°C during 24 hr, whereafter they were photographed and the diameter of the clear zones was measured with ImageJ. From the hen egg white analyses, standard curves of lysozyme activity were obtained to calculate the hemolymph lysozyme concentration.

### 2.5 Statistical analyses

To analyze if larval density, host plant species, and their interaction have an effect on the different immune functions in *S. littoralis*, separate general linear models were performed for each immune function measurement with density (high or low), plant species (cotton, cabbage, or maize), and their interaction as explanatory variables. For all analyses, the partial eta squared effect sizes were estimated. The PO activity and protein content were square root transformed, and lysozyme activity was logarithmized to obtain normality. One outlier was excluded from the analysis of PO activity. All analyses were performed in JMP version 14Pro.

## 3 RESULTS

Density was found to significantly affect the degree of cuticular melanization (Table 1), where individuals in the high‐density treatment were scored as darker than solitary larvae (means ± *SE*: low density: 3.864 ± 0.102, high density: 5.339 ± 0.106). There was a tendency that diet affected the cuticular melanization, where maize fed larvae tended to be more pale (means ± *SE*: cotton: 4.755 ± 0.117, cabbage: 4.591 ± 0.124, maize: 4.314 ± 0.139, Table 1). The interaction between diet and density did, however, not have any significant effect on the cuticular melanization.

**TABLE 1:** The effects of host plant diet, larval density, and their interaction on immune traits. Results and partial eta squared effect sizes from general linear models. Significant effects are highlighted in bold

Explanatory variable	Cuticular melanization	Encapsulation, area	Encapsulation, melanization score	Sqrt PO activity	Sqrt protein content	LOG lysozyme activity
Diet	*F*_2,127_ = 2.535 *p* = .083 ŋ2*p* = .034	*F*_2,99_ = 1.213 *p* = .302 ŋ2*p* = .025	*F*_2,99_ = 0.325 *p* = .724 ŋ2*p* = .007	*F*_2,115_ = 3.005 *p* = .054 ŋ2*p* = .048	*F*_2,119_ = 0.320 *p* = .727 ŋ2*p* = .005	***F*_2,102_ = 3.329** ***p* = .040** **ŋ2*p* = .061**
Density	***F*_1,127_ = 95.251** ***p* < .0001** **ŋ2*p* = .438**	*F*_1,99_ = 1.113 *p* = .294 ŋ2*p* = .011	*F*_1,99_ = 0.065 *p* = .800 ŋ2*p* = .001	***F*_1,115_ = 9.790** ***p* = .002** **ŋ2*p* = .078**	*F*_1,119_ = 0.035 *p* = .852 ŋ2*p* < 0.001	*F*_1,102_ = 0.035 *p* = .853 ŋ2*p* < 0.001
Diet*Density	*F*_2,127_ = 1.671 *p* = .192 ŋ2*p* = .027	*F*_2,99_ = 0.339 *p* = .714 ŋ2*p* = .007	*F*_2,99_ = 1.245 *p* = .293 ŋ2*p* = .026	*F*_2,115_ = 0.491 *p* = .614 ŋ2*p* = .008	*F*_2,119_ = 2.417 *p* = .094 ŋ2*p* = .040	*F*_2,102_ = 2.282 *p* = .108 ŋ2*p* = .042

When analyzing the artificial encapsulation assay, there were no significant effects on neither the encapsulated area nor the degree of melanization (Table 1).

**FIGURE 2:**
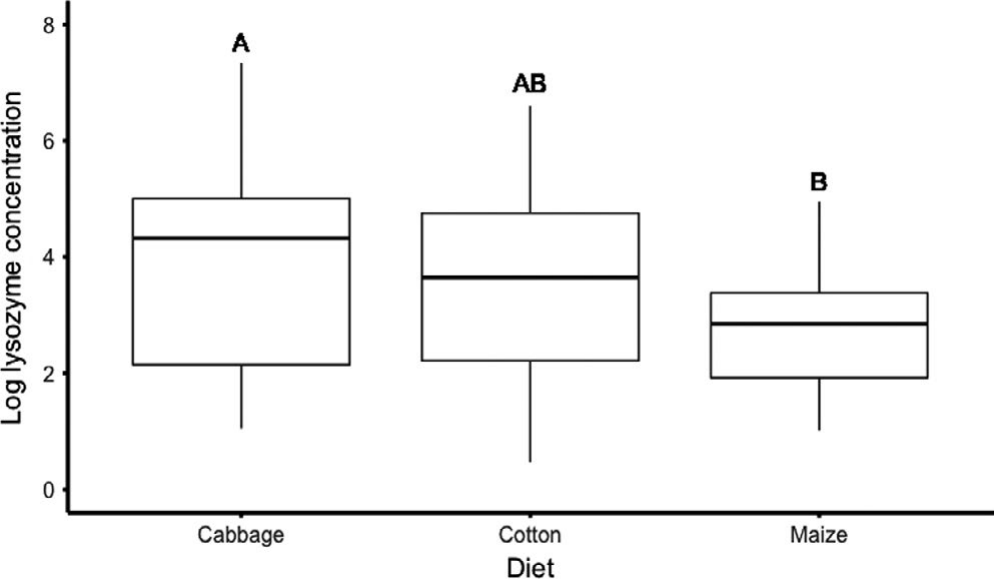
The effect of host plant diet on lysozyme activity in larval hemolymph. Boxes indicate 25th and 75th percentiles, lines within boxes indicate medians, and lines within boxes indicate medians, and whiskers include values within 1.5 times the interquartile range. Different letters above the bars indicate significant differences between treatments (*p* < .05), results from general linear model (Table 1), *N* = 103

In the hemolymph immune assays, the PO activity was significantly affected by density, where larvae in crowded conditions had a higher PO activity (Figure 2a, Table 1). Furthermore, there was a tendency that PO activity was affected by larval diet where individuals that had fed maize had a higher enzyme activity than individuals fed cabbage or cotton (Figure 2b, Table 1). There was, however, no interaction effect of diet and density on PO activity (Table 1). There was a tendency that the interaction between diet and density affected hemolymph protein content (Table 1) but there were no significant differences between either main factors alone for this response variable; thus, protein content was not used to correct for condition in any of the other analyses (Table 1). Lysozyme activity was found to be significantly affected by host plant diet, where cabbage fed larvae had the highest antibacterial activity and maize fed larvae the lowest activity (Figure 2, Table 1). However, neither density nor the interaction between density and diet affected lysozyme activity (Table 1).

## 4 DISCUSSION

Here, the potential for host plant species to mediate a density‐dependent prophylactic immune investment in larvae of the polyphagous moth *S. littoralis* was investigated. There were differences found in immune function both between density treatments and between host plant species but no support for interacting effects of these factors. Interestingly, the results indicated that different plant species may elicit stronger effects on different functions of the immune system. These findings may be relevant for the understanding of host plant selection in this species, but also for biological control of polyphagous pests.

The cuticular melanization was darker, and PO activity was increased in high larval densities while lysozyme activity and encapsulation traits did not differ between densities. That not all immune functions are upregulated in a crowded condition could be due to trade‐offs between different functions within the immune system (Sadd & Schmid‐Hempel, 2009) and is something that has been seen previously too for *S. littoralis* (Cotter, Hails, et al., 2004). In the current study, there were no significant effects from the interaction between diet and density on the immune responses. This indicates that the potential for host plant diet to mediate the density‐dependent prophylaxis is low, even if the effect size for the lysozyme activity was slightly larger. The lack of interacting effects may be surprising considering the importance of diet for mounting an immune response. The cost of immune responses may, however, often go undetected (see discussion in Bartlett et al., 2018; Dallas et al., 2016) and could be expressed first in later life stages, for example, during reproduction (Sheldon & Verhulst, 1996). Thus, differences depending on host plant diet between individuals reared in high and low densities may be evident only in other life‐history traits. Although there were no differences in hemolymph protein content between plant species in the current study, *S. littoralis* larvae have in general a poor development on maize (Rösvik et al., 2020). This indicates that maize would be the host plant of overall lowest quality where an increased prophylactic investment could be most challenging, (see e.g., Kirschman et al., 2017), and where potential costs of a prophylactic investment might be seen in later life stages or in other traits. In the current study, neither survival during the experiment nor larval weight at the point of the immune assays were measured. This could otherwise have been important factors to consider when interpreting the results. In future research, it may be interesting to look into how the actual nutritional profiles of different host plants affect insect immune responses as well as if increased immune responses have host plant‐specific trade‐offs with, for example, growth, survival, and reproduction.

The different host plant species did, however, in themselves affect the larval immune responses. Larvae that were fed cabbage and cotton had a higher lysozyme activity than larvae fed maize, and there were tendencies that cabbage‐ and cotton‐reared larvae also had darker cuticula than maize‐reared larvae. This further indicates that the low quality of maize as a host plant for *S. littoralis* (see e.g., Rösvik et al., 2020) also has negative impact on larval immunity. On the other hand, there were tendencies that maize fed larvae had higher PO activity than larvae on the other diets, which often indicates an increased immune function against, for example, fungal infections (Dubovskiy et al., 2013). As PO is an enzyme which is involved in melanin production (Gonzalez‐Santoyo & Cordoba‐Aguilar, 2012), the high levels of circulating PO in the hemolymph together with the relatively pale cuticula in larvae reared on maize indicates that there are other limiting factors in maize diet that hinders melanin production. Having high levels of circulating PO in the hemolymph in the absence of infection may be deleterious for insects as the PO could oxidize to toxic quinonoids (Sugumaran & Barek, 2016). Thus, although maize‐reared individuals may be more protected from endoparasites they could also suffer from low protections against ectoparasites and from having toxic waste‐products in the hemolymph.

Although some of the effects of plant diet were only tendencies and should be interpreted with caution before they are confirmed with a larger sample size, it is interesting to note that they are in line with previous studies that have shown that the components of the immune system in *S. littoralis* responds differently depending on the specific blend of nutrients in the diet (Cotter et al., 2011). Experiments that manipulated both the nutrients (quality) and the calories (quantity) in an artificial diet revealed that while PO activity was found to be mostly affected by dietary carbohydrates, lysozyme activity was more dependent on protein content of the diet (Cotter et al., 2011). In a natural context, the nutrient composition of a given host plant species may thus be better suited for one or the other of the immune functions. This means that host plants may indeed enhance the immune response of *S. littoralis* but also that there is a specificity between host plant species and particular immune responses and that a specific plant is not an overall immune‐enhancing diet. Moreover, as the immune functions are defenses against different kinds of antagonists, host plants may provide larvae with better or worse protection depending on which type of immune response that is needed to defeat the antagonist. For example, the current results indicate that it would be beneficial for larvae to develop on maize if they are exposed to fungal infections, but maize would not provide them with a good immune response if they are exposed to bacterial infections.

Addressing the effects that host plant species have on insect immune function as a measure of larval performance could therefore be important for our understanding of polyphagous host plant selection, where female preference and offspring performance is not always correlated (Gripenberg et al., 2010). For example, a mixture of different host plant species is optimal for general performance for the polyphagous moth *Parasemia plantaginis* but specific host plants are beneficial for immunity of the insect (Ojala et al., 2005). Moreover, immune function in *Lobesia botrana* is increased when the insect develops on alternative host plant species and the variation in how different host plant species affect insect immune function has thus been suggested to maintain polyphagy (Muller et al., 2015). *Spodoptera littoralis* has a complex host plant selection which depends on both innate preference hierarchies between plant species and plastic preference induction from earlier host plant experience (Anderson et al., 2013; Proffit et al., 2015; Thöming et al., 2013), where female preference is not correlated with offspring performance (K. Karlsson Green, C. de Pasqual, M. Litto, P. Anderson, unpublished data). For example, although larval performance is poor on maize (Rösvik et al., 2020), females have a high preference for this plant species (Thöming et al., 2013). Potentially, such preference may be maintained if a poor host plant in the event of a specific disease actually enhances offspring survival. This scenario has been shown in the monarch butterflies, *Danaus plexippus,* where one of its milkweed host plant species increases adult lifespan when butterflies are infected with a parasite but decreases adult lifespan when butterflies are uninfected (Sternberg et al., 2012). The host plant‐mediated effects on immune function could therefore be one of the missing links in our understanding of the relationship between preference and performance in generalist species, as well as of the maintenance of polyphagy.

That different host plant species allow stronger responses in different components of the insect immune system may also be of importance for sustainable pest management, especially if some host plants are beneficial for the insect against pathogens and other host plants against parasitoids. Sustainable management of pest insects is often performed with biological control, such as parasitoids or pathogens, and the efficiency of these biocontrol agents may thus vary between different host plant species. This has been reported from *Spodoptera frugiperda*, where soybean genotype affected baculovirus infectivity (Shikano et al., 2017; Shikano et al., 2017), and in *Malacosoma disstria,* which was a 100 times more resistant to *Bacillus thuringiensis* on aspen than on sugar maple (Kouassi et al., 2001). These findings thus emphasize the need of fundamental research in ecology and evolution to fine‐tune the control strategies in sustainable pest management (Karlsson Green et al., 2020). For example, for some crops, or crop varieties, parasitoids may be more efficient and for others pathogens may be better to use due to the interaction between host plant species and pest immune function.

## 5 CONCLUSION

In summary, the current study confirmed previous findings of density‐dependent prophylaxis in the polyphagous crop pest moth *S. littoralis*. This prophylactic induction was, however, not mediated by different host plant species. Instead, host plant species could in itself affect the immune response, and different plants elicited increased responses in different immune functions. These results indicate that plant species differences in nutrient content and chemistry may be more or less valuable for certain immune responses and that insects thus may be more or less protected against different kinds of antagonists on different host plant species.

## CONFLICT OF INTEREST

The author declares no conflict of interests.

## AUTHOR CONTRIBUTION

**Kristina Karlsson Green:** Conceptualization (lead); Data curation (lead); Formal analysis (lead); Funding acquisition (lead); Investigation (lead); Methodology (lead); Project administration (lead); Resources (lead); Software (lead); Validation (lead); Visualization (lead); Writing‐original draft (lead); Writing‐review & editing (lead).

### DATA AVAILABILITY STATEMENT

The data is deposited at Dryad https://doi.org/10.5061/dryad.6hdr7sr14.
